# Emotional exhaustion among operating room and general ward nurses: evidence from a regional hospital in Taiwan

**DOI:** 10.3389/fpubh.2025.1648696

**Published:** 2025-09-17

**Authors:** Li Li, Chih-Hsuan Huang, Yii-Ching Lee, Hsin-Hung Wu

**Affiliations:** ^1^International College, Guangzhou College of Commerce, Guangzhou, China; ^2^Hubei Enterprise Culture Research Center, Hubei University of Economics, Wuhan, China; ^3^Business School, Hubei University of Economics, Wuhan, China; ^4^Department of Health Business Administration, Hungkuang University, Taichung, Taiwan; ^5^Department of Business Administration, National Changhua University of Education, Changhua, Taiwan; ^6^Faculty of Education, State University of Malang, Malang, East Java, Indonesia

**Keywords:** emotional exhaustion, operating room, general ward, nurses, regional hospital

## Abstract

This study examined differences in emotional exhaustion (EE) among nurses working in operating rooms (ORs) and general wards (GWs) at a regional teaching hospital in Taiwan. A total of 263 nurses completed the EE subscale of the Chinese Safety Attitudes Questionnaire (CSAQ). Results showed that OR nurses consistently reported significantly higher EE levels than GW nurses, especially regarding fatigue, emotional depletion, and interpersonal strain. Among GW nurses, older and mid-career individuals exhibited significantly higher EE scores, suggesting an age-related cumulative burden. Independent samples *t*-tests and ANOVA were applied to identify group differences. Gender, educational attainment, and managerial role were not significantly associated with EE levels. These findings highlight the combined impact of clinical unit context and individual demographics on nurse wellbeing. Healthcare organizations are advised to implement targeted interventions, such as stress debriefing for OR teams and long-term support for mid-career or senior GW nurses, to reduce EE and promote psychological resilience.

## 1 Introduction

Burnout syndrome, particularly emotional exhaustion (EE), has emerged as a critical concern in healthcare due to its detrimental effects on the physical and psychological wellbeing, job performance, and patient safety of healthcare professionals (1). EE, defined as a state of extreme emotional depletion and fatigue, is widely recognized as the core dimension of burnout in healthcare contexts (1, 2). Healthcare workers, especially nurses, are frequently exposed to high levels of EE, largely due to the emotionally demanding nature of their roles, which require sustained interpersonal interactions with patients and multidisciplinary teams (3). Extensive research has demonstrated that higher levels of EE among nurses are significantly associated with an increased incidence of medical errors, lower patient satisfaction, higher infection rates, and greater turnover intentions (4). Moreover, EE adversely impacts nurses' job satisfaction and significantly reduces the quality of patient care, thus potentially endangering patient safety (5).

In Taiwan, nurses frequently contend with heavy workloads and elevated occupational stress, in part due to unfavorable staffing levels. According to recent data from the Ministry of Health and Welfare, nurse-to-patient ratios in general wards typically range from 1:9 to 1:12—significantly exceeding the internationally recommended standard of 1:6 (6). Such staffing imbalances place considerable strain on nursing personnel, increase task density, and heighten the risk of emotional exhaustion (7). To systematically monitor nurses' EE and support patient safety efforts, the Joint Commission of Taiwan (JCT) developed the Chinese version of the Safety Attitudes Questionnaire (CSAQ), which integrates the EE dimension from the Maslach Burnout Inventory–Human Services Survey (MBI-HSS). This tool is utilized annually in hospitals across Taiwan to assess the levels of EE among healthcare staff (8). Previous studies have also confirmed a significant negative correlation between nurses' EE and patient safety culture, identifying EE as a critical indicator of healthcare quality (5).

Nevertheless, despite the abundance of research on nurses' EE, most studies have treated nurses as a homogeneous group, seldom exploring potential differences in EE across specific clinical units. In practice, nursing roles and work environments vary considerably across hospital departments. For example, nurses working in operating rooms (ORs) are frequently subjected to high-pressure situations that demand precise judgment, rapid decision-making, and close teamwork under stringent time constraints. Conversely, nurses in general wards (GWs) operate in relatively stable but emotionally taxing environments characterized by continuous patient care and administrative responsibilities (9). These differences are not merely procedural but carry distinct psychological implications. In ORs, nurses face acute stressors driven by surgical complexity, interprofessional coordination, and the high stakes of intraoperative decision-making, which may lead to rapid emotional depletion. In contrast, GW nurses encounter more chronic, cumulative stress arising from prolonged caregiving, frequent patient-family interactions, and workload predictability that can mask burnout risks until later stages. Understanding such unit-specific stress profiles is vital to designing targeted interventions. For instance, structured debriefings may be more suitable in ORs, while long-term mentorship and emotional resilience programs may be more effective in GWs. By tailoring strategies to each unit's operational reality, healthcare systems can improve nurse wellbeing, reduce turnover, and ultimately enhance patient safety and care continuity.

Given these contextual differences, it is imperative to investigate whether significant disparities in EE exist between OR and GW nurses. Addressing this research gap is essential for developing unit-specific interventions aimed at mitigating EE, thereby enhancing both nurse wellbeing and patient safety. Accordingly, this study seeks to empirically examine the differences in EE between OR and GW nurses in a regional hospital in Taiwan, contributing valuable insights for healthcare management and policy development.

## 2 Relevant literature

### 2.1 EE and burnout syndrome

Burnout syndrome was first conceptualized by Freudenberger (10) and has since received widespread attention in healthcare due to its profound impacts on healthcare workers' job performance, wellbeing, and patient safety. Maslach and Jackson (1) defined burnout as comprising three core dimensions: EE, depersonalization, and diminished personal accomplishment, with EE considered the central and most influential component. EE specifically refers to feelings of emotional depletion and extreme fatigue resulting from continuous psychological and emotional demands (1, 2).

Previous studies have consistently reported that EE negatively affects job satisfaction, increases turnover intentions, and contributes to decreased quality of patient care (5). For example, Getie et al. (11) found that nurses' EE is significantly associated with reduced job satisfaction, increased burnout, and a heightened risk of patient safety incidents. In their systematic review, Quesada-Puga et al. (12) further confirmed the close association between EE, work disengagement, and reduced quality of patient care, particularly within high-pressure units. Similarly, Kwon et al. (13) reported a significant association between emotional labor-induced EE and increased medical errors and turnover intentions among nurses. Studies conducted within the healthcare context in Taiwan have also corroborated these findings, revealing that EE among nurses is significantly associated with higher medical error rates, lower patient satisfaction, and overall deterioration of healthcare quality (8).

### 2.2 Measurement of EE: MBI-HSS and CSAQ

The Maslach Burnout Inventory–Human Services Survey (MBI-HSS), developed by Maslach and Jackson (1), remains the most widely used instrument for assessing burnout and EE. The MBI-HSS evaluates the severity and frequency of burnout symptoms through items such as “I feel emotionally drained from my work” and “I feel used up at the end of the workday” (1). In recent years, the instrument has been extensively validated, demonstrating robust psychometric properties. For example, Lin et al. (14) conducted a cross-cultural psychometric study of the MBI-HSS Medical Personnel version (MBI-HSS-MP) and confirmed its stability and validity across different languages and cultural contexts. Similarly, Al Mutair et al. (15) validated the applicability of the MBI-HSS within the Saudi Arabian healthcare system, confirming its strong discriminant validity, particularly for the EE and depersonalization dimensions.

In response to the growing need to systematically monitor patient safety culture in hospitals, the Joint Commission of Taiwan (JCT) adapted the Safety Attitudes Questionnaire (SAQ) originally developed by Sexton et al. (16) and introduced the Chinese version, known as the Chinese Safety Attitudes Questionnaire (CSAQ), in 2007. The CSAQ has undergone several revisions, and in 2014, the JCT incorporated the EE dimension from the MBI-HSS to more comprehensively assess healthcare professionals' emotional wellbeing and its influence on patient safety (7). The current CSAQ consists of eight dimensions, including teamwork climate, safety climate, job satisfaction, perceptions of management, working conditions, stress recognition, EE, and work-life balance. Specifically, the EE dimension contains nine items (see Table 1) directly adapted from the MBI-HSS, enabling hospitals in Taiwan to systematically and comprehensively assess nurses' burnout levels and emotional states within the framework of patient safety culture (5, 8).

**Table 1:** Nine questions of EE from the CSAQ.

**No**.	**Description**
EE 1	I feel like I'm at the end of my rope
EE 2	I feel burned out from my work
EE 3	I feel frustrated by my job
EE 4	I feel I'm working too hard on my job
EE 5	I feel emotionally drained from my work
EE 6	I feel used up at the end of the workday
EE 7	I feel fatigued when I get up in the morning and have to face another day on the job
EE 8	Working with people all day is really a strain for me
EE 9	Working with people directly puts too much stress on me

### 2.3 Consequences of EE

The adverse consequences of EE among nurses have been extensively documented. Research has shown that nurses with higher levels of EE often experience deteriorations in healthcare quality, increased medical error rates, reduced job satisfaction, and elevated turnover intentions (5). Kwon et al. (13) further reported that EE among nurses is strongly associated with medical errors, work-related stress, and turnover intentions, and that this relationship persists across different hospital levels. Moreover, the study by Labrague and Nwafor (17) found significant associations between EE and leadership styles, deteriorating psychological wellbeing, increased absenteeism, and higher turnover rates, reflecting the profound impact of EE on nurses' workplace adaptation and retention intentions. More specifically, nurses experiencing severe EE are more likely to exhibit indifferent or negative care behaviors, which undermine patient trust and satisfaction with nursing services (18). Additionally, studies have indicated that EE increases the emotional labor burden on nurses, reduces their empathy, and diminishes their engagement in patient safety culture, further threatening the quality of healthcare delivery (19).

From the perspective of physical and psychological health, EE has been found to contribute to deteriorating wellbeing among nurses, leading to reduced work efficiency, diminished commitment to patient care, and significantly increased absenteeism rates (20). Cho and Steege (21), in their systematic review, also highlighted that EE is closely associated with fatigue, which not only increases absenteeism and medical error occurrences but also negatively affects patient care outcomes. Furthermore, a meta-analysis by Li et al. (20) confirmed that EE is moderately to highly correlated with lower patient satisfaction, deteriorating healthcare quality, and increased turnover intentions among nurses, underscoring the urgent need for healthcare institutions to address and intervene in nurses' EE.

## 3 Methods

### 3.1 Measure and data sample

This study aimed to examine the differences in EE perceptions among nurses working in different clinical units, specifically ORs and GWs, in a regional teaching hospital in Taiwan. A cross-sectional quantitative design was employed using a survey method. Convenience sampling was adopted to recruit registered nurses from ORs and GWs during the data collection period in 2020. The EE of nurses was measured using nine questions of the EE dimension from the CSAQ (see Table 1), which was developed and validated by the JCT. Each question is assessed using a 5-point Likert scale ranging from 1 (strongly disagree) to 5 (strongly agree), where higher scores indicate greater levels of fatigue or burnout, reflecting poorer recovery from EE. Ethical approval for this study was obtained from the Institutional Review Board (IRB) of the regional teaching hospital (approval no. HP190028). Participation in the survey was considered as providing implied consent for the use of the data.

### 3.2 Data analysis

To explore and analyze the differences in EE perceptions between nurses working in ORs and GWs, this study conducted a systematic statistical analysis. Data were processed and analyzed using SPSS version 25.0. Initially, descriptive statistics were used to summarize the demographic characteristics of the participants and the score distributions of the nine EE items from the CSAQ. Subsequently, independent samples *t*-tests were conducted to compare the mean scores of the nine EE items between ORs and GWs, aiming to examine whether significant differences in EE perceptions existed between the two groups. Furthermore, for professional background variables with three or more categories, one-way analysis of variance (ANOVA) was applied to further explore their potential influence on the scores of the nine EE items and to identify any significant differences among the various categories.

### 3.3 Sample size and statistical power

To ensure that our sample was adequate to detect meaningful differences, we conducted a *post hoc* power analysis using G^*^Power version 3.1. Based on the actual sample sizes (OR = 60; GW = 203), an alpha level of 0.05, and the observed effect size for the overall EE score (Cohen's *d* = 0.78), the statistical power (1–β) was calculated to be 0.9996. This indicates that the study was sufficiently powered to detect statistically significant differences between groups.

## 4 Results

### 4.1 Demographic information

Table 2 presents the demographic characteristics of nurses working in ORs and GWs. A total of 60 OR nurses and 203 GW nurses participated in the study. In terms of gender, female nurses predominated in both groups, with a higher proportion in GWs (93.6%) compared to ORs (81.7%). The proportion of male nurses was relatively higher in ORs (18.3%) than in GWs (6.4%). Regarding age, nurses aged 21–30 years accounted for the largest proportion in both groups; however, this was more pronounced in GWs (60.6%) than in ORs (38.3%). OR nurses showed a higher proportion of older staff aged 41–50 years (25.0%) and 51–60 years (10.0%) compared to their GW counterparts (13.7% and 2.5%, respectively). In terms of position, more nurses in GWs held supervisory or managerial roles (11.3%) compared to those in ORs (5.0%). Concerning organizational tenure, both groups were distributed across various experience levels, with nurses having 5–10 years of experience being the most common in both ORs (35.1%) and GWs (25.6%). The proportion of nurses with over 21 years of experience was similar in both groups (8.3% in ORs and 8.9% in GWs). In terms of education, nearly all participants in both settings held a college or university degree (98.3% in ORs and 98.5% in GWs), while only a small fraction reported having a senior high school education. Overall, the data indicate that nurses in ORs tend to be older, include a higher proportion of males, and have longer tenure, whereas nurses in GWs are generally younger, predominantly female, and more likely to hold supervisory positions.

**Table 2:** Characteristics of nurses in ORs and GWs.

**Demographic variables**	**ORs (*****n*** = **60)**		**GWs (*****n*** = **203)**	
	**Frequency**	**Percentage**	**Frequency**	**Percentage**
**Gender**				
Male	11	18.3	13	6.4
Female	49	81.7	190	93.6
**Age**				
21–30 years old	23	38.3	123	60.6
31–40 years old	16	26.7	47	23.2
41–50 years old	15	25.0	28	13.7
51–60 years old	6	10.0	5	2.5
**Managerial position**				
Yes	3	5.0	23	11.3
No	57	95.0	180	87.7
**Experience in organization**				
1–11 months	3	5.0	13	6.4
1–2 years	12	20.0	43	21.2
3–4 years	11	18.3	42	20.7
5–10 years	21	35.1	52	25.6
11–20 years	8	13.3	35	17.2
21 years or more	5	8.3	18	8.9
**Education**				
Senior high school	1	1.7	3	1.5
College/University	59	98.3	200	98.5

### 4.2 Comparison of the EE questions in ORs and GWs

Table 3 presents the results of independent samples *t*-tests and corresponding Cohen's d values for the nine EE items, comparing nurses in operating rooms (ORs) and general wards (GWs). OR nurses reported significantly higher mean scores across all items (all *p* < 0.001), with effect sizes ranging from moderate to large (Cohen's *d* = 0.54–1.03), indicating not only statistical significance but also meaningful practical differences. Among the items, the most substantial disparities were observed in EE7 (“I feel fatigued when I get up in the morning…”; *d* = 1.02), EE5 (“I feel emotionally drained from my work”; *d* = 1.03), and EE8 (“Working with people all day is really a strain for me”; *d* = 0.94). These items reflect anticipatory fatigue, emotional depletion, and interpersonal strain—hallmarks of the OR environment. The pronounced effect sizes suggest that high-intensity shift work, complex surgical procedures, and sustained multidisciplinary coordination contribute to the elevated emotional burden among OR nurses. The remaining items (EE1–EE4, EE6, EE9) also showed significant between-group differences with moderate effect sizes (Cohen's *d* = 0.54–0.85), reinforcing the overall pattern of greater EE in the OR setting. These results underscore the cumulative impact of task complexity, time pressure, and patient safety responsibilities on OR nurses' psychological wellbeing.

**Table 3:** Comparison of nine questions between Ors and GWs using independent samples *t*-tests.

**EE questions**	**ORs**		**GWs**		***t***	***p*-*value***	**Cohen's *d***
	**Mean**	**SD**	**Mean**	**SD**			
EE 1	3.73	1.13	3.12	1.05	3.75	< 0.001	0.57
EE 2	3.53	1.19	2.67	1.02	5.08	< 0.001	0.81
EE 3	3.90	1.08	3.10	0.90	5.22	< 0.001	0.85
EE 4	3.07	0.80	2.59	0.92	3.90	< 0.001	0.54
EE 5	3.77	1.14	2.72	0.98	6.44	< 0.001	1.03
EE 6	3.23	1.20	2.57	0.96	3.92	< 0.001	0.65
EE 7	3.90	1.26	2.70	1.01	6.75	< 0.001	1.12
EE 8	4.12	1.12	3.21	0.94	5.72	< 0.001	0.93
EE 9	4.10	1.13	3.36	0.92	4.64	< 0.001	0.76
Overall EE	3.71	1.12	2.89	0.97	2.99	0.004	0.78

EE1–EE9 represents the nine emotional exhaustion (EE) questionnaire items.

### 4.3 Comparison of bivariable analysis of demographic in ORs and GWs

This study used ANOVA to explore whether OR nurses' perceptions of EE, measured across nine items, varied significantly by demographic characteristics such as age, organizational tenure, and educational attainment (see Table 4). A significant difference in EE5 (“I feel emotionally drained from my work”) was observed across age groups (*p* = 0.003), with the highest scores reported by nurses aged 31–40 [M = 4.43, SD = 0.62; 95% CI (4.13, 4.72)] and 51–60 [M = 4.50, SD = 0.83; 95% CI (3.68, 5.31)], indicating that mid-career and older nurses experienced greater EE. While EE7, EE8, and EE9 also showed variation by age, these did not reach statistical significance (*p* = 0.053–0.073). Organizational tenure was significantly associated with several EE items. Nurses with 5–10 years of experience reported notably higher levels of exhaustion than those with less than one year. Specifically, for EE1 (“I feel like I'm at the end of my rope”), the 5–10 year group scored M = 4.04 [SD = 1.07; 95% CI (3.63, 4.44)] compared to M = 2.00 [SD = 1.73; 95% CI (−0.13, 4.13)] in the 1–11 month group (*p* = 0.023). Similar differences were observed in EE5 [M = 4.28 vs. 2.33; 95% CI (3.86, 4.69) vs. (1.27, 3.39); *p* = 0.050], EE8 [M = 4.71 vs. 3.00; 95% CI (4.38, 5.05) vs. (2.22, 3.78); *p* = 0.032], and EE9 [M = 4.76 vs. 2.33; 95% CI (4.49, 5.02) vs. (1.27, 3.39); *p* < 0.001]. These findings suggest that emotional strain increases with prolonged clinical exposure, and that both age and tenure are important contributors to perceived EE among OR nurses.

**Table 4:** Demographic variables and EE dimensions in operating room nurses.

**Demographic variables\EE dimensions**	**EE1 Mean (SD)**	**EE2 Mean (SD)**	**EE3 Mean (SD)**	**EE4 Mean (SD)**	**EE5 Mean (SD)**	**EE6 Mean (SD)**	**EE7 Mean (SD)**	**EE8 Mean (SD)**	**EE9 Mean (SD)**
**Age (** ***n*** **)**									
21–30 years (23)	3.43 (1.23)	3.26 (1.28)	3.69 (1.18)	3.00 (0.85)	3.39 (1.26)	3.04 (1.46)	3.56 (1.30)	3.82 (1.07)	3.73 (1.32)
31–40 years (16)	4.06 (0.85)	4.00 (1.03)	4.37 (0.71)	3.25 (0.93)	4.43 (0.62)	3.50 (1.09)	4.50 (0.96)	4.62 (0.71)	4.56 (0.72)
41–50 years (15)	3.60 (1.24)	3.26 (1.22)	3.60 (1.24)	2.80 (0.56)	3.33 (1.04)	2.93 (0.88)	3.53 (1.35)	3.80 (1.42)	3.93 (1.09)
51–60 years (6)	4.33 (0.81)	4.00 (0.63)	4.16 (0.75)	3.50 (0.54)	4.50 (0.83)	4.00 (0.63)	4.50 (0.83)	4.66 (0.81)	4.67 (0.81)
*p-value*	0.184	0.145	0.144	0.220	0.003^**^	0.190	0.053	0.054	0.073
*Post-hoc*					31–40> 21–30> 41–50				
**Gender (** ***n*** **)**									
Male (11)	3.72 (1.34)	3.81 (1.32)	3.90 (1.30)	2.54 (0.52)	3.81 (1.32)	3.54 (1.21)	4.00 (1.26)	4.18 (1.16)	4.09 (1.44)
Female (49)	3.73 (1.09)	3.46 (1.15)	3.89 (1.04)	3.18 (0.80)	3.75 (1.10)	3.16 (1.19)	3.87 (1.26)	4.10 (1.12)	4.10 (1.06)
*p-value*	0.407	0.406	0.371	0.295	0.926	0.884	0.858	0.775	0.252
**Education (** ***n*** **)**									
Senior high school (1)	5.00 (n/a)	4.00 (n/a)	5.00 (n/a)	3.00 (n/a)	5.00 (n/a)	3.00 (n/a)	5.00 (n/a)	5.00 (n/a)	5.00 (n/a)
College/University (59)	3.71 (1.13)	3.52 (0.75)	3.00 (1.08)	3.06 (0.80)	3.75 (1.13)	3.23 (1.20)	3.88 (1.26)	4.10 (1.12)	4.08 (1.13)
*p-value*	0.263	0.695	0.310	0.934	0.279	0.846	0.383	0.432	0.427
**Experience in organization (** ***n*** **)**									
1–11 months (3)	2.00 (1.73)	2.66 (0.57)	3.00 (1.73)	2.66 (0.57)	2.33 (1.15)	2.33 (1.15)	3.00 (0.81)	3.00 (0.74)	2.33 (1.15)
1–2 years (12)	3.83 (1.11)	3.33 (1.55)	3.75 (1.13)	3.00 (1.04)	3.66 (1.15)	2.83 (1.52)	3.41 (1.56)	3.75 (1.13)	3.58 (1.31)
3–4 years (11)	3.90 (0.83)	3.45 (0.93)	4.00 (0.89)	3.09 (0.70)	3.63 (1.28)	3.63 (1.02)	3.90 (1.30)	4.09 (1.04)	4.18 (0.98)
5–10 years (21)	4.04 (1.07)	4.14 (0.96)	4.28 (0.95)	3.19 (0.81)	4.28 (1.01)	3.71 (1.05)	4.42 (0.92)	4.71 (0.78)	4.76 (0.62)
11–20 years (8)	3.75 (1.03)	3.12 (1.24)	3.62 (1.40)	3.12 (0.64)	3.62 (0.91)	2.62 (0.91)	3.87 (1.45)	3.87 (1.45)	4.12 (0.99)
21 years or more (5)	2.80 (0.83)	2.80 (0.83)	3.40 (0.54)	2.80 (0.83)	3.20 (0.83)	2.80 (0.84)	3.40 (1.14)	3.60 (1.34)	3.40 (1.14)
*p-value*	0.023^*^	0.056	0.252	0.868	0.050^*^	0.057	0.163	0.032^*^	0.000^***^
*Post-hoc*	5–10 years> 1–11 months				5–10 years> 1–11 months			5–10 years> 1–11 months	5–10 years> 1–11 months
**Managerial position (** ***n*** **)**									
Yes (3)	2.33 (1.52)	2.66 (1.15)	3.00 (1.73)	2.66 (1.15)	3.00 (1.73)	2.33 (1.52)	4.00 (1.00)	4.33 (1.15)	3.33 (2.08)
No (57)	3.80 (1.07)	3.57 (1.17)	3.94 (1.04)	3.08 (0.78)	3.80 (1.09)	3.28 (1.17)	3.89 (1.27)	4.10 (1.12)	4.14 (1.07)
*p-value*	0.522	0.760	0.174	0.321	0.247	0.773	0.149	0.694	0.572

^*^p < 0.05, ^**^p < 0.01, ^***^p < 0.001.

EE1–EE9 represents the nine emotional exhaustion (EE) questionnaire items

Analysis of GW nurses showed that age and organizational tenure were significantly associated with certain dimensions of EE (see Table 5). Specifically, EE7 (“I feel fatigued when I get up in the morning...,” *p* = 0.029) and EE8 (“Working with people all day is really a strain for me,” *p* = 0.027) differed significantly across age groups. *Post hoc* comparisons revealed that nurses aged 51–60 reported significantly higher scores on EE7 [M = 3.80, SD = 1.09; 95% CI (2.84, 4.75)] and EE8 [M = 4.20, SD = 1.30; 95% CI (3.01, 5.38)] than those aged 21–30 [EE7: M = 2.58, SD = 0.96; 95% CI (2.42, 2.75); EE8: M = 3.16, SD = 0.89; 95% CI (3.01, 3.31)], and 41–50 [EE7: M = 2.75, SD = 0.92; 95% CI (2.43, 3.06); EE8: M = 2.96, SD = 0.96; 95% CI (2.58, 3.33)]. These findings suggest that older nurses may experience greater fatigue and interpersonal strain due to prolonged clinical demands and accumulated job stressors. Similarly, tenure-related differences were found in EE1 (“I feel like I'm at the end of my rope,” *p* = 0.027), with nurses in the 5–10 year group reporting higher exhaustion levels [M = 3.09, SD = 1.03; 95% CI (2.79, 3.39)] than those with less than 1 year of experience [M = 2.53, SD = 1.05; 95% CI (1.89, 3.16)]. Although EE7–EE9 also varied numerically across tenure groups, these differences were not statistically significant (*p* = 0.13–0.18). Overall, the results suggest that EE among GW nurses is shaped primarily by structural factors such as age and tenure.

**Table 5:** Demographic variables and EE dimensions in GW nurses.

**Demographic variables\EE dimensions**	**EE1 Mean (SD)**	**EE2 Mean (SD)**	**EE3 Mean (SD)**	**EE4 Mean (SD)**	**EE5 Mean (SD)**	**EE6 Mean (SD)**	**EE7 Mean (SD)**	**EE8 Mean (SD)**	**EE9 Mean (SD)**
**Age (** ***n*** **)**									
21–30 years (123)	3.13 (1.10)	2.67 (1.03)	3.04 (0.92)	2.68 (0.89)	2.68 (0.96)	2.56 (0.92)	2.58 (0.96)	3.16 (0.89)	3.27 (0.95)
31–40 years (47)	3.10 (1.04)	2.70 (1.06)	3.27 (0.82)	2.48 (1.03)	2.78 (1.04)	2.72 (1.01)	2.87 (1.09)	3.36 (0.91)	3.55 (0.82)
41–50 years (28)	3.03 (0.83)	2.67 (0.90)	3.10 (0.87)	2.42 (0.79)	2.75 (0.96)	2.35 (0.98)	2.75 (0.92)	2.96 (0.96)	3.25 (0.84)
51–60 years (5)	3.40 (0.89)	2.40 (1.14)	2.60 (0.89)	2.20 (0.83)	2.80 (1.30)	2.69 (1.14)	3.80 (1.09)	4.20 (1.30)	4.20 (1.30)
*p-value*	0.908	0.942	0.286	0.313	0.930	0.461	0.029^*^	0.027^*^	0.064
*Post-hoc*							51–60 years> 21–30 years	51–60 years> 41–0 years	
**Gender (** ***n*** **)**									
Male (13)	3.07 (1.32)	2.23 (0.92)	2.61 (0.96)	2.07 (1.11)	2.30 (1.10)	2.15 (0.80)	2.38 (1.04)	2.76 (0.72)	2.92 (0.86)
Female (190)	3.12 (1.03)	2.70 (1.02)	3.13 (0.88)	2.62 (0.89)	2.74 (0.97)	2.60 (0.96)	2.72 (1.00)	3.23 (0.94)	3.38 (0.92)
*p-value*	0.268	0.955	0.421	0.062	0.154	0.336	0.684	0.184	0.257
**Education (** ***n*** **)**									
Senior high school (3)	2.66 (0.57)	2.67 (0.58)	2.67 (0.58)	2.00 (1.00)	2.00 (1.00)	2.00 (1.00)	2.67(0.58)	2.67 (0.58)	2.67 (0.58)
College/University (200)	3.12 (1.05)	2.68 (1.02)	3.10 (0.89)	2.60 (0.91)	2.73 (0.98)	2.58 (0.95)	2.70 (1.01)	3.21 (0.93)	3.37 (0.92)
*p-value*	0.345	0.271	0.577	0.768	0.740	0.672	0.272	0.431	0.325
**Experience in organization (** ***n*** **)**									
1–11 months (13)	2.53 (1.05)	2.61 (0.96)	2.61 (1.04)	2.46 (1.05)	2.53 (0.87)	2.53 (0.87)	2.46 (0.87)	2.84 (0.98)	3.07 (0.95)
1–2 years (43)	3.48 (1.16)	2.76 (1.04)	3.09 (0.92)	2.69 (0.98)	2.76 (0.99)	2.55 (0.88)	2.74 (0.97)	3.34 (0.78)	3.39 (0.90)
3–4 years (42)	2.90 (0.98)	2.59 (1.06)	3.00 (0.98)	2.59 (0.82)	2.61 (0.96)	2.47 (0.91)	2.52 (0.96)	3.07 (0.97)	3.16 (1.03)
5–10 years (52)	3.09 (1.03)	2.69 (1.03)	3.28 (0.77)	2.76 (0.80)	2.71 (0.95)	2.67 (0.96)	2.63 (1.02)	3.19 (0.84)	3.32 (0.80)
11–20 years (35)	3.02 (0.95)	2.60 (1.01)	3.02 (0.78)	2.40 (1.03)	2.68 (0.99)	2.57 (1.00)	2.77 (1.03)	3.11 (0.96)	3.48 (0.85)
21 years or more (18)	3.38 (0.91)	2.78 (1.00)	3.27 (0.95)	2.27 (0.89)	3.05 (1.16)	2.55 (1.24)	3.27 (1.07)	3.66 (1.23)	3.77 (1.06)
*p-value*	0.027^*^	0.961	0.181	0.272	0.684	0.962	0.136	0.130	0.185
*Post-hoc*	5–10 years > 1–11 months								
**Managerial position (** ***n*** **)**									
Yes (23)	3.04 (1.14)	2.52 (1.08)	3.04 (0.82)	2.00 (0.95)	2.47 (1.03)	2.26 (0.96)	2.82 (1.19)	3.47 (1.16)	3.69 (1.05)
No (180)	3.12 (1.04)	2.69 (1.01)	3.10 (0.90)	2.66 (0.89)	2.75 (0.97)	2.61 (0.95)	2.68 (0.98)	3.17 (0.90)	3.31 (0.89)
*p-value*	0.397	0.339	0.637	0.527	0.328	0.786	0.165	0.235	0.140

^*^p < 0.05.

EE1–EE9 represents the nine emotional exhaustion (EE) questionnaire items.

## 5 Discussion

### 5.1 Clinical unit differences in EE

This study conducted a comparative analysis of EE among nurses working in ORs and GWs at a regional teaching hospital in Taiwan. Findings showed that OR nurses consistently reported significantly higher average scores across all nine EE items in the CSAQ, suggesting a higher overall emotional burden. The largest disparities appeared in EE7, EE5, and EE8, highlighting the cumulative impact of high-pressure, time-sensitive, and decision-intensive environments characteristic of surgical units. These results extend prior research by Shah et al. (4), who emphasized heightened burnout risks in acute care settings, and are aligned with Quesada-Puga et al. (12), who identified fast-paced decision-making and interprofessional complexity as key contributors to emotional strain. Notably, while previous studies often report gender differences in burnout, our findings revealed no significant EE variation by gender across both units, suggesting the overwhelming effect of clinical demands may override gender-based differences in this particular setting. From a workforce planning perspective, higher EE among OR nurses imply a need for targeted structural support, such as scheduled debriefing sessions, team-based workload redistribution, and policy-level attention to OR staff wellbeing. These interventions could not only mitigate chronic stress but also improve retention rates and enhance care quality in high-intensity departments. Future research should evaluate the long-term efficacy of such interventions and examine how organizational reforms can reduce burnout risk across diverse clinical contexts.

### 5.2 Demographic predictors of EE

In both GWs and ORs, EE was significantly associated with age and tenure, revealing clear demographic vulnerability patterns. Nurses aged 51–60 in GWs reported higher scores on EE7, while their OR counterparts aged 31–40 and 51–60 showed the highest levels of emotional drain on EE5. These patterns suggest that cumulative clinical exposure—especially among mid-career and senior staff—intensifies emotional burden over time. Similarly, tenure-related differences indicated that nurses with 5–10 years of experience, regardless of unit, reported elevated exhaustion across several items (e.g., EE1, EE5, EE8, EE9), supporting Huang et al.'s (5) argument that burnout risk often peaks during transitional mid-career stages when responsibilities intensify but institutional support may lag. These findings extend the work of Ahmed et al. (18) and Huang et al. (5), reinforcing the role of age and tenure as critical factors in occupational burnout.

Notably, our data did not reveal significant EE differences by gender, education, or supervisory status, contrasting with literature linking burnout to gender disparities. This divergence may reflect the overriding impact of structural and environmental stressors—such as clinical intensity and task complexity—over individual demographics, as also suggested by Ahmed et al. (18). From an organizational perspective, these insights point to the importance of targeted interventions such as structured debriefing sessions for OR teams, mentorship programs for mid-career nurses, and early warning systems to detect burnout trajectories. Such strategies may enhance emotional resilience, reduce turnover risk, and improve long-term care quality across diverse clinical units.

### 5.3 Work characteristics as predictors of EE

From the perspective of task structure, OR nurses consistently operate in high-risk, time-sensitive environments that demand rapid decision-making and seamless interprofessional coordination. Their duties—ranging from preoperative preparation to sterile equipment management and intraoperative contingency handling—require sustained cognitive and emotional engagement. These occupational pressures are reflected in the significantly elevated EE scores observed among OR nurse, particularly on items related to fatigue and interpersonal strain. These findings extend prior research by Sonoda et al. (9) and Quesada-Puga et al. (12), who identified high task density and decision-making complexity as critical drivers of emotional burnout in surgical teams. Additionally, systemic factors such as high patient loads and irregular shifts further compound the psychological burden in OR settings.

In contrast, nurses in GWs typically work in more routine-based environments. However, our results indicate that mid-career and senior nurses in GWs reported higher levels of EE, especially in items associated with fatigue and emotional depletion. This suggests that prolonged exposure to emotionally intense caregiving, coupled with limited professional growth, may gradually erode resilience. These findings align with Huang et al. (5), who proposed an “accumulative exhaustion trajectory” among experienced staff, and echo the work of Quesada-Puga et al. (12) on organizational vulnerability among unsupported senior nurses. Importantly, our analysis found no significant EE differences by gender, education, or supervisory role—contrary to previous studies—highlighting that structural and task-related stressors may override individual demographic factors in shaping burnout outcomes. To mitigate these risks, we recommend implementing structured debriefing sessions for OR teams, mentorship programs for mid-career staff, and system-level reforms that improve workload balance, mental health resources, and promotion pathways.

## 6 Conclusion

This study provides empirical evidence that EE among nurses varies significantly across clinical contexts and is influenced by demographic factors. OR nurses exhibited significantly higher EE than GW nurses, reflecting the intensified psychological burden associated with time-sensitive procedures, multidisciplinary coordination, and complex patient care. Additionally, age and organizational tenure were particularly associated with EE in ward nurses, suggesting that prolonged clinical exposure may contribute to accumulated emotional fatigue. These findings highlight the need for targeted interventions that address the specific stressors of each clinical setting. For example, structured debriefing sessions for OR teams and mentorship programs for mid-career or senior nurses could help alleviate chronic stress and promote coping resilience. At the organizational level, such initiatives may contribute not only to enhanced staff wellbeing but also to improved patient care quality, better staff retention, and more sustainable healthcare workforce performance. Future research should extend this work by employing longitudinal and intervention-based designs to assess the effectiveness of tailored support programs and institutional policies in reducing EE and fostering psychological resilience across diverse healthcare environments. However, this study has several limitations. The cross-sectional design precludes causal inference, and self-reported data may be subject to bias. Additionally, the findings are based on a single regional hospital, which may limit generalizability. Future studies should adopt longitudinal, multi-site designs to validate and extend these results.

## Data Availability

The original contributions presented in the study are included in the article/supplementary material, further inquiries can be directed to the corresponding authors.
